# Are female directors more employee-friendly? Board gender diversity and employee benefits: evidence from China

**DOI:** 10.3389/fpsyg.2024.1285056

**Published:** 2024-07-31

**Authors:** Yao Liu, Yingkai Tang, Yunfan Yang

**Affiliations:** ^1^Business School, Sichuan University, Chengdu, China; ^2^Sichuan Tianfu Bank, Chengdu, China

**Keywords:** board gender diversity, female directors, employee benefits, monetary incentives, nonmonetary rewards

## Abstract

The imperative of gender diversity in corporate governance and the adoption of a human-centric governance paradigm are intensifying globally. The structure of board directors, key influencers to corporate decisions, notably shape policies, crucially in emerging markets like China where gender issues are still evolving. Therefore, employing a penal dataset comprising 8,973 firm-year observations from publicly A-share-listed Chinese firms spanning 2006 to 2021, this study empirically examines the impact of board gender diversity on the responsiveness to both employee monetary incentives and non-monetary rewards. The findings unveil a positive correlation, indicating an augmentation in per-employee compensation and an increased likelihood of implementing non-monetary programs, including stock-ownership plans, retirement benefits, and occupational safety certification, in the presence of higher board gender diversity. Notably, these positive associations are more accentuated in state-owned firms, as well as those with lower executive compensation and diminished institutional ownership. Our results remain consistent after considering robustness as well as endogeneity. This empirical evidence not only contributes robust statistical support to the ongoing global initiatives advocating for gender diversity in corporate governance but also underscores the efficacy of boards of directors in effectively managing stakeholder interests, particularly in fostering employee-friendly practices within emerging markets like China.

## 1 Introduction

The composition of corporate boards has long been an important issue in corporate governance research (Kirsch, 2018). Appointing female directors tends to lead to more diverse board composition, but such appointments can affect the nature of board processes and outcomes (Terjesen et al., 2009), as well as the leadership styles of female directors that differ from those of their male counterparts (Sila et al., 2016), like female leaders were more likely to pay individual attention to diverse employees concerning their empathy and cultural consideration (Kolpakov and Boyer, 2021), which in turn affect a firm’s policymaking, operational outcomes, and long-term development. However, according to a variety of data, males occupy the vast majority of corporate directorships around the world, and female directors are significantly underrepresented (Kirsch, 2018). We note that women held 31.3% of total director seats in developed markets as constituents of the MSCI World Index. In contrast, only 15.9% of board seats among constituents of the MSCI Emerging Markets Index were held by females (MSCI, 2022). But here’s the interesting thing: A 2018 Harris Poll reveals that 50% of Americans prefer to work for a female leader since female-led companies are more purpose-driven and more likely to offer childcare and equal pay (Cameron, 2018). Why should we care about employee opinions and manage them? because Haver Analytics and Citi Research find that labor costs account for over 60% of total business costs. Corporate human capital investment is therefore a critical business decision for top executives (Fan et al., 2021). There is no doubt that human capital is the key competency and vital source of competitive advantage (Wright and Perrone, 1977; Lippman and Rumelt, 1982; Coff, 1997). Enhancing salary and other non-monetary benefits, employer culture and image, training and development programs, and the work environment can greatly improve employee satisfaction (Xu et al., 2020), while higher employee satisfaction can not only stimulate innovative thinking (Manso, 2011; Acharya et al., 2014), and improve employee productivity (Sauermann and Cohen, 2010) but also prompt employees to switch their perspective of their efforts from short-term performance to long-term value (Flammer and Kacperczyk, 2016), which ultimately manifests itself as an increase in the innovation output of the firms (Xu et al., 2020). In other words, higher employee satisfaction and better employee relationship maintenance play a vital role in building corporate resilience and sustainable development.

On the one hand, investments in employees can directly promote the ability to attract and retain talent, enhance job satisfaction, foster a positive work environment, and build an efficient workforce (Tunyi et al., 2023). For instance, Zingales (2000) argues that superior employee family benefit programs can positively influence employee satisfaction, which in turn increases employee retention and motivation to benefit shareholders. Profit-sharing is usually seen as an indicator that a firm will be concerned with employees’ overall welfare (Charness and Kuhn, 2007), and firms that award profit-sharing to employees have higher levels of employee satisfaction (Creek et al., 2019). This is consistent with prior research showing that profit-sharing can influence performance via its effects on employee attitudes (Coyle-Shapiro et al., 2002). Edmans (2011) investigated the “100 Best Companies to Work for in America” and found a positive and strong relationship between a company’s employee satisfaction and its long-term stock performance. On the other hand, employee welfare is one of the corporate social responsibility (CSR) activities that board members can use their decision-making authority to improve, such as providing higher quality employee benefits and maintaining better employment policies for disabled workers (Ning et al., 2017), while the implementation of CSR activities increases firm value and crisis response ability (Malik, 2015) through various ways, such as improving employee productivity (Valentine and Fleischman, 2008), risk management (Dhaliwal et al., 2011), and earnings quality (Kim et al., 2012). Also, the most proximal and impactful context influencing employee motivation and behavior is the team within which employees often work (Chen and Kanfer, 2024). Indeed, work teams typically provide both the cues and consequences for a team member’s behavior, as well as exerting many other social- and technical-based influences that manifest in work organization (Mathieu et al., 2019). On the flip side, without proper relationship maintenance, employee turnover weakens a company’s competitiveness (Aime et al., 2010), accentuates its weaknesses (Campbell et al., 2012), and places a company in a precarious position that seriously impairs its capacity to respond to crises and its resilience to recover from them (Lengnick-Hall et al., 2011) due to the transfer complementary resources and opportunities leaving with the exiting employees’ (Agarwal and Helfat, 2009), in particular supporting team members (Groysberg et al., 2007) and social networks (Burton and Beckman, 2007). However, turnover can be greatly reduced if firms provide employees with both monetary and non-monetary benefits (Williams and Livingstone, 1994), as these employee incentives increase employee satisfaction, autonomy, and commitment (Raelin, 1994), which in turn create strong internal bonds and loyalty to the firm and better motivate these employees to stay and perform well (Dess and Shaw, 2001; Piva and Vivarelli, 2005).

However, there is a significant difference in employee policies between female and male directors (Creek et al., 2019; Nadeem et al., 2020). However, leadership is responsible for analyzing the views, norms, values, and organizational culture that are transmitted within the company and that influence employee behavior in every way (Pérez, 2019). Firms with diverse boards are more likely to adopt programs that signal organizational support for employees and benevolence which foster a higher level of job satisfaction (Creek et al., 2019). In terms of leadership style, researchers often characterize female managers by high levels of inclusion, communication, communal values, and knowledge sharing (Powell et al., 2008; Lyngsie and Foss, 2017). Drawing from upper echelons and social role theoretical viewpoints, Kirsch (2018) and Tunyi et al. (2023) contend that women on boards are likely to be more sensitive, sympathetic, tolerant, supportive, compassionate, and ethical in their decision-making, especially toward employment-related issues, than men. From a philanthropic and CSR perspective, firms with female directors might introduce or sustain better employee benefits policies and practices (e.g., generous benefits, and initiatives to promote a healthy work-life balance; Byron and Post, 2016; Creek et al., 2019; Chen and Kao, 2022) as they are less likely to have a business background and are more likely to have experience in philanthropic and community service than males (Hillman et al., 2002). Additionally, according to incongruity models (Heilman and Okimoto, 2007; Del Carmen Triana et al., 2024), the stereotypical belief that women are more communal (e.g., caring, sociable) does not match with the qualities people believe are required for leaders, whereas the stereotypic belief that men are more agentic (e.g., assertive, competitive) coincides with beliefs about leadership requirements (Koenig et al., 2011; Heilman, 2012). Consequently, women are burdened with a perceived lack of fit, resulting in difficulties when striving to acquire leadership positions (Nater et al., 2023). Therefore, female directors may provide stakeholder-focused views in response to a crisis or to shape business strategy (Hillman et al., 2002; Nadeem et al., 2020) due to the bottlenecks and obstacles in their promotion process. The “hander-than-men” promotion makes them prone to offer more training opportunities to other potential employees (Byron and Post, 2016), and also work harder to maintain their boardroom status through employee-friendly policies.

Research on the impact of board gender diversity has flourished in recent years, but three research gaps remain. Firstly, the impact of board gender diversity on employee incentives, including monetary incentives and non-monetary rewards, has been largely overlooked. The sections discussing the benefits to employees mostly concentrate on LGBT-friendly policy-making (Cook and Glass, 2014; Everly and Schwarz, 2015), a decreased likelihood of downsizing (Tunyi et al., 2023), better CSR performance (Cumming et al., 2015; Byron and Post, 2016; Nadeem et al., 2020), a more flexible working environment (Olsen et al., 2017), and cash-profit sharing (Rekker et al., 2014). However, in a recent study about gender and response to incentives, Sittenthaler and Mohnen (2020) indicate that men exhibit significantly higher performance when motivated by monetary incentives as opposed to non-monetary ones. Conversely, women’s performance is notably higher in response to non-monetary incentives, attributed to feelings of appreciation and perceived performance pressure within a tournament setting. This study effectively unveiled gender preferences in response to various incentives, but it approached the topic from the perspective of policy recipients. Specifically, Sittenthaler and Mohnen (2020) did not explore the reactions of policymakers, such as board members, toward employee benefits, not even the heterogeneous responses of policymakers toward monetary and non-monetary benefits. Secondly, the conclusions of the above studies on the impact of board gender diversity are mixed. Most of the literature argues that higher board gender diversity is beneficial to corporate governance (Liu, 2021; Bennedsen et al., 2022; Kuzey et al., 2022), but there are also some neutral or contrary to the influence of gender on managerial effectiveness. For example: found that both positive and negative dimensions of corporate social responsibility are unrelated to gender diversity when there is a token female representation on the boards of directors. Thirdly, the research mentioned above also mostly concentrates on developed markets. There is scant research on how gender diversity affects employee benefits in emerging markets like China. Therefore, in this study, our major goal is to determine whether having a more gender-diverse board (i.e., having more female directors) has an impact on the monetary incentives and non-monetary rewards given to employees in China.

This study seeks to fill the gap and makes three important contributions. First, it enriches the literature on the impact of board gender diversity and provides empirical evidence. We study the association between female representation on boards and the monetary incentives as well as non-monetary rewards of employees using a sample of China’s A-share listed firms in the Shanghai and Shenzhen stock markets over the period from 2006 to 2021. We measure board gender diversity as the Shannon index and the percentage of female directors on the boards. Based on 8,973 firm-year observations, the empirical results show that the more diverse gender construct on boards increased the employee average salary and the likelihood of the implementation of non-monetary benefits such as stock ownership plans, retirement benefits, and occupational safety certification. We also find this positive relationship is more pronounced in state-owned firms as well as in situations with lower executive compensation and lesser institutional ownership. Our results remain consistent when considering robustness as well as endogeneity. Therefore, this study contributes to existing research on gender diversity in employee-friendly policies in emerging markets like China and enriches research on firm heterogeneity in the context of gender diversity.

Second, this study supplements existing literature on employee relationship management (REM). As employees are one of the stakeholders of a firm, the high employee costs and the severe consequences of employee exit have made REM an important topic in the field of corporate governance (Fan et al., 2021). However, prior research on employee-friendly policies tends to focus on stock-ownership plans, retirement plans, etc. (Dhanesh, 2014), and there is a paucity of research on the influencing factors of employee compensation and overall non-monetary benefits. Therefore, this study empirically explores the effect of board diversity on average employee salary as well as the implementation of the overall non-monetary programs (We collect data about eight non-monetary programs. They are stock-ownership plans, retirement benefits, safety management systems, safety generation training, occupational safety certification, employee vocational training, employee communication channels, and other non-monetary programs). This provides empirical evidence on the employee policy preferences and employee friendliness of female directors.

Third and last, our results can provide certain guidance and empirical evidence for firms to nominate and appoint female directors to increase the overall diversity of the board. Appropriate monetary incentives and non-monetary rewards contribute significantly to good employee satisfaction, relations, and productivity, while a highly skilled and committed workforce is crucial to improving the firm’s performance and value, as well as its sustainability and resilience to respond to a crisis. By increasing the overall level of board gender diversity, it is possible to demonstrate more employee friendliness in this regard. This study provides empirical evidence for the different leadership styles and policy perspectives of female directors relative to their male counterparts. Corporate boards need more diverse perspectives.

The study is structured as follows: The next section summarizes prior literature and develops our research hypotheses. The third section describes the data and methodology. The fourth section presents the empirical results, and a conclusion follows.

## 2 Literature review and hypotheses development

### 2.1 Board gender diversity and employee benefits

On the one hand, female directors are able to bring different perspectives to the board compared to male directors (Daily et al., 2000; Rose, 2007; Kim and Starks, 2016). Specifically, socialization theory suggests that females have been socialized to be more nurturing, compassionate, focused on developing interpersonal skills, and cooperative (Zelezny et al., 2000; Tunyi et al., 2023). Therefore, the females will create a more participative, democratic, and communal leadership style on the board (Ben-Amar et al., 2017), and their different leadership abilities from male directors will also facilitate more informed and smarter decisions. One of them is using “low cost” reforms such as raising employee compensation to achieve much higher employee retention, productivity, and innovation, as well as better company reputation and performance (Eagly and Carli, 2003). Research has found that compensation serves as a signal of an organization’s values and culture (Kuhn, 2009), increasing employees’ wages can largely increase their loyalty (Cronqvist et al., 2009), and higher-paid employees are less likely to leave their jobs relative to those who are lower-paid (Campbell et al., 2012). This effect is even more pronounced among high-performing employees, as Trevor et al. (1997) found a strong moderating effect of wage growth rate on turnover among high-performing employees, largely due to the greater focus on reward inequity among those employees (Gerhart and Milkovich, 1992). Turban and Keon (1993) find that pay raises based on individual performance made an organization more attractive compared to seniority-based raises, and this effect varies according to the individual’s need for fulfillment. It is worth emphasizing that performance at higher job levels is more expensive and difficult to replace, and it tends to have a larger impact on the success and sustainability of the company (Trevor et al., 1997).

On the other hand, female directors are more likely to “please” employees through direct policy changes (Kolpakov and Boyer, 2021) such as salary increases to strengthen their position on the board, as they need to spend more effort on status-building than male directors. Specifically, first, it is so hard to become a female director. Even when women attain the professional prerequisites, their access to board seats is much stricter than that of men due to organizational barriers including non-transparent recruitment and unequal pay (Bilimoria and Piderit, 1994; Smith and Parrotta, 2018). Token theory (Kanter, 1977; Smith and Parrotta, 2018) also explains why there is a low probability of hiring a second female on the board following the appointment of the first. Second, even after becoming a female director, the decision-making authority of female directors is much weaker relative to that of males, and female directors are more likely to become a symbol or be responsible for corporate affairs of relatively lesser importance. Token theory explains that the first female on the board is purposefully appointed to represent the minority (women) rather than to contribute toward decision-making through their knowledge, competence, and experience (Kanter, 1977; Smith and Parrotta, 2018; Guldiken et al., 2019), i.e., employing a few female directors is not because of their potential but simply in response to institutional pressures (Smith and Parrotta, 2018).

With this background, Radu and Smaili (2022) suggest that boards with a critical mass of members from demographically underrepresented groups are especially likely to advocate for progressive practices such as pay growth which foster positive employee attitudes and satisfaction. In addition, as female representation on boards increases, so does female influence on the corporate decision-making process (Elstad and Ladegard, 2010), making female directors more likely to implement policies that are consistent with their values, leadership style, or status entrenchment. Therefore, this leads us to our first hypothesis:

*Hypothesis 1a*: Firms with more females on the board are more likely to have better employee monetary benefits.

Beyond the regular salary incentives, non-monetary rewards, such as employee insurance, profit-sharing plans, and training opportunities, are also even more attractive to female directors. We argue that female directors on the board may cast additional non-monetary reward programs for three reasons.

First, female directors are likely to be more sensitive, sympathetic, tolerant, supportive, and empathetic toward employment-related issues than males (Tunyi et al., 2023). Therefore, they will provide more comprehensive employee benefits based on salary increases, especially when these non-monetary programs are relatively cost-effective and cycle-shorter. Second, since female directors are more likely to have a philanthropic and community service background than males (Hillman et al., 2002), this enables them to pay more attention to the needs of employees beyond salaries, such as a healthy work-life balance, flexible working environment, and subsidized childcare, etc.) (Byron and Post, 2016; Creek et al., 2019; Chen and Kao, 2022). Third, some literature argues that women are risk-averse (Lai et al., 2021). However, investments in human capital like employees are risky (Lai et al., 2021) and are more risky than investments in physical capital, in part because the rewards of investments in personnel competencies are less unpredictable (Fan et al., 2021). Therefore, female directors may favor these non-monetary forms of employee benefits more to reduce the risk of overall employee-friendly policies but equally as a complement to lower employee compensation as well as to preserve the same level of job satisfaction. Consequently, the following hypothesis is proposed:

*Hypothesis 1b*: Having more females on the board helps with the implementation of non-monetary employee incentives.

### 2.2 Moderating conditions

Firms with various characteristics were differently affected by the board gender diversity. For instance, as opposed to non-state-owned firms (Non-SOE), state-owned firms (SOE) in China have to assume greater social responsibilities for both economic and non-economic goals (Jiang et al., 2021). Therefore, state-owned firms are more likely to have managers who opt for higher CSR performance and even sacrifice some economic profit (Zu and Song, 2009; Li and Zhang, 2010). In addition, generally speaking, state-owned firms have a relatively larger organization size and more comprehensive governance systems, which make them more capable of implementing non-monetary rewards than non-state-owned firms.

Executive compensation is an incentive alignment mechanism that harmonizes the interests of shareholders and the management (Holmstrom and Milgrom, 1991; Tosi and Gomez-Mejia, 1994; Radu and Smaili, 2022), one of which is the trade-off between fulfilling social responsibilities and maximizing economic profits (Derchi et al., 2021). Higher executive compensation can greatly reduce the conflicts and agency costs between management and shareholders and also make them more likely to lead the firm in line with the interests of shareholders which may reduce the investment in employees. In addition, it has also been shown that the pay gap between executives and rank-and-file employees leads to power asymmetries in the workplace, where executives view lower-level employees as dispensable and unworthy of human dignity (Desai et al., 2010). A study of employee complaints and executive compensation indicated that the more a company pays its executives, the higher its overall meanness score for mistreating employees (Cai et al., 2011).

By holding a large and stable stake in a company, institutional investors may endeavor to extract their own private profits through excessive intervention in corporate governance (Edmans, 2011), such as employee-related policymaking, which may increase information asymmetry and even exacerbate conflicts (Buchanan et al., 2018) between the firm and its non-investment stakeholders like employees. At the same time, such excessive intervention may also reduce management’s incentives and integrity (Guiso et al., 2015), which ultimately leads to a reduction in CSR implementation as well as lower performance. In addition to corporate governance, institutional investors also specialize in monitoring activities (Buchanan et al., 2018). When institutional holdings are greater, their monitoring role creates formalized institutions within the firm, and this can be far more effective than informal institutions such as empathy and CSR culture, making it more difficult to implement employee-friendly policies for personal cultural preferences. For which the following hypothesis is proposed:

*Hypothesis 2*: The positive impacts of board gender diversity on employee benefits vary due to differences in ownership structures, executive compensation, and institutional ownership.

**Figure 1:**
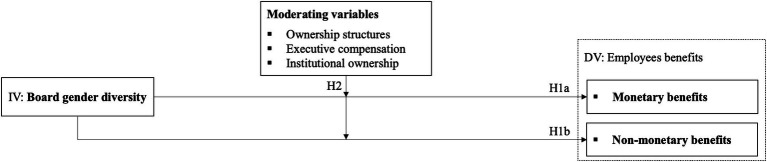
Research framework.

Figure 1 summarizes our hypotheses and serves as a research framework for this paper. Through this research framework, we aim to provide insight into the impact of board gender diversity on employee benefits and examine the role of various firm-level characteristic moderators in this relation. This contributes to a more comprehensive understanding of how board gender diversity affects corporate management and employee relations, providing insights for future research and business practice.

## 3 Data and methodology

### 3.1 Data

Due to the imperfect and unstrict information disclosure regulation, Chinese listed firms disclosed less data on the gender of board members before 2006. Therefore, based on the availability of gender data, the data samples for this study are China’s A-share listed firms in the Shanghai and Shenzhen stock markets between 2006 and 2021. For the data collection, the Chinese Research Data Service Platform (CNRDS) and China Stock Market & Accounting Research (CSMAR) Database contain firm-level financial information and individual-level information on corporate board members, which are among the most widely accepted and most important databases in the field of management and economics in China due to the breadth and accuracy of their data collection (Bao and Li, 2024; Xu et al., 2024). Specifically, our key outcome variables are monetary and non-monetary benefits to employees, which were collected from the CNRDS database. Gender data and other firm-level financial and corporate governance data, such as financial ratios, firm characteristics, and governance structures were collected from the CSMAR Database. To make our sample more representative, this study screened it to the following basic principles:

1. Due to the special nature of the business model in the financial sector, we removed the financial firms. Specifically, according to the China Securities Regulatory Commission (CSRC) Guidelines on Industry Classification of Listed Companies (revised in 2012), four major categories, including monetary and financial services, capital market services, insurance, and other financial industries, were removed from the financial sector.
2. Excluding specially treated firms from the original sample, i.e., removing firms marked ST, *ST, PT, etc. from the sample, to avoid anomalies in financial performance and governance data.
3. The firms with any missing value were eliminated.

After the above screening, our final sample consists of 8,973 firm-year observations.

### 3.2 Variables

#### 3.2.1 Independent variable: board gender diversity

We followed prior studies to measure our main independent variable, that is, gender diversity on the board, as the Shannon index (Campbell et al., 2012; Nadeem et al., 2020; Liu, 2021; Kuzey et al., 2022). We also applied an alternative proxy measure as a robustness check: the proportion of female directors to total directors on boards (*FemaleRatio*) (Arayakarnkul et al., 2022). The Shannon index is calculated as


$$\mathrm{ShannonIndex}=-\overset{2}{\underset{i=1}{\sum }}P_{i}\ln P_{i}$$

Where *i* = (1, 2) is the number of gender categories (i.e., male and female), and $P_{i}$ is the proportion of each category on the board. When the number of male and female directors is closer, the *ShannonIndex* is larger. *ShannonIndex* takes the maximum value of 0.6931 when the ratio of males to females is the same.

#### 3.2.2 Dependent variable: employee benefits

Based on our hypotheses, our dependent variable, employee benefits, is composed of two parts: monetary benefits (*Monetary*) and non-monetary benefits (*NonMonetary*). Specifically, we measure the monetary benefits as the logarithm of compensation per employee of the company (including cash paid) (Kim and Jang, 2020; Bennedsen et al., 2022), which was calculated as (compensation paid to employees + compensation payable to employees at the end of the natural year - compensation payable to employees at the beginning of the natural year) / total number of employees (unit: RMB). For non-monetary rewards, we collected information on the following eight non-monetary programs in terms of data availability (Kong et al., 2024): stock-ownership plans, retirement benefits, safety management systems, safety generation training, occupational safety certification, employee vocational training, employee communication channels, and other non-monetary programs except the above seven programs. *NonMonetary* equals 1 when the firm executes one of these non-monetary programs, 2 when it implements two, etc. *NonMonetary* is a number between [0, 8] as a result. The strict distinction between different programs here also facilitates the heterogeneity analysis of the different non-monetary rewards in the further test.

#### 3.2.3 Control variables

In reference to previous studies on gender diversity and employee benefits (Campbell and Mínguez-Vera, 2008; Nadeem et al., 2020; Fan et al., 2021), we use firm size (*Size*), debt level (*Leverage*), return on assets (*ROA*), ownership structure (*Property*), shares balance (*SharesBalance*), the combination of CEO and Chairman roles (*Duality*), board independence (*Independence*), year (*YearDum*) and industry (*IndustryDum*) as the control variables.

To be more specific, first, for financial information, larger firms and those with higher ROA may have more resources and financial stability, allowing them to offer more comprehensive employee benefits packages. Highly leveraged firms may allocate a significant portion of their earnings to debt servicing, limiting the funds available for employee benefits. Second, for corporate governance details, if the largest shareholder is state-owned, it may indicate a level of stability and government backing. This could lead to more job security and potentially better employee benefits due to the perceived stability of state-owned firms. The distribution of shares among the top shareholders and the duality between the CEO and Chairman can influence the decision-making power within the company. A separation of these shares and roles may lead to more checks and balances, potentially benefiting employees. A more independent board might prioritize long-term sustainability and employee welfare over short-term gains. Also, boards with diverse perspectives may consider a broader range of employee benefits, reflecting the needs of different stakeholders. Last, considerations related to the year and industry. The economic landscape of a given year may impact a company’s fiscal health, wherein prosperous periods may encourage more favorable employee benefits, while economic downturns may necessitate cost-cutting measures. Divergent industries may uphold distinct norms concerning employee benefits, with technology companies, for instance, emphasizing stock options, while manufacturing enterprises focus on conventional benefits like healthcare and retirement plans. To mitigate the influence of economic cycles and industry characteristics, dummy variables for the year and industry are incorporated into the regression model. Table 1 gives the definition and measures for all the variables.

**Table 1:** Definitions of variables.

	Variables	Definition and measure
Independent variable	ShannonIndex	The Shannon index is calculated as $-\overset{2}{\underset{i=1}{\sum }}P_{i}\ln P_{i}$ , where P*i* has the same meaning as in the previous expression.
	FemaleRatio	Female ratio, the proportion of female directors to total directors on boards
Dependent variable	Monetary	Log-transformed per-employee compensation of the company (including cash paid).
	NonMonetary	*NonMonetary* = 1 when the firm implements any of the previously mentioned non-monetary programs, *NonMonetary* = 2 when it implements two of them, and so on. That is, *NonMonetary* is an integer taking from [0, 8].
Moderating variables	ExeCompensation	Logarithm of total executive compensation, excluding allowances received by directors, supervisors, and executives
	Institutional	Institutional investor shareholding (%)
Control variables	Size	Logarithm of total assets
	Leverage	Asset liability ratio = total liabilities / total assets
	ROA	total net profit / total assets
	Property	The nature of the ownership structure. 1 if the largest shareholder is state-owned; otherwise, 0
	SharesBalance	2nd-5th largest shareholder shareholding / 1st largest shareholder shareholding
	Duality	1 if the Chief Executive Officer (CEO) and Chairman are the same person, otherwise 0
	Independence	The ratio of independent directors to the number of whole directors
	YearDum	Dummy variable, the natural year in which the company is located
	IndustryDum	Dummy variable, based on the industry classification issued by the CSRC Guidelines in 2012

### 3.3 Model specification

To examine the impact of board gender diversity on employee benefits, we estimate the following baseline model:


$$\begin{array}{c} \begin{array}{l} \left(Non\right)\mathrm{Monetar}y_{i,t}=\beta_{0}+\beta_{1}\mathrm{ShannonInde}x_{i,t}+\beta_{2}Size_{i,t}+\\ \beta_{3}\mathrm{Leverag}e_{i,t}+\beta_{4}ROA_{i,t}\; \end{array}\\ \begin{array}{l} +\beta_{5}\mathrm{Propert}y_{i,t}+\beta_{6}\mathrm{SharesBalanc}e_{i,t}+\\ \beta_{7}\mathrm{Dualit}y_{i,t}+\beta_{8}\mathrm{Independenc}e_{i,t} \end{array}\\ +\overset{n}{\underset{i=1}{\sum }}\mathrm{YearDum}+\overset{n}{\underset{i=1}{\sum }}\mathrm{IndustryDum}+\varepsilon_{i,t} \end{array}$$

Where *i* denotes firm and *t* denotes the year, $\varepsilon_{i,t}$ is the random error term. $\left(Non\right)\mathrm{Monetar}y_{i,t}\text{,}$ and $\mathrm{ShannonInde}x_{i,t}$ are the dependent variable and independent variable of this study, respectively. The meanings of control variables are explained in “Table 1 Definitions of Variables.” We also included the industry-fixed and year-fixed effects to control for macroenvironmental shocks and time-invariant industry traits. The coefficient we are interested in is $\beta_{1}$ , which estimates the impact of board gender diversity on employee benefits. A positive and significant $\beta_{1}$ suggests that a more gender-diverse board exerts a positive effect on employee benefits, while a negative and significant $\beta_{1}$ indicates that higher gender diversity pushed employee benefits down. It is worth noting that due to the different data structures and distributions of $\mathrm{Monetar}y_{i,t}$ and $\mathrm{NonMonetar}y_{i,t}$ , this study adopts the Ordinary Least Squares (OLS) model and the Poisson model for regression analysis, respectively (Atif et al., 2021). We also winsorized the top and bottom 1% of all the continuous variables from their distributions to conform to other studies and avoid the interference of outliers on the results (Hendricks and Singhal, 2005). The standard errors are clustered at the firm level to control for heteroscedasticity and within-firm correlation in the residuals (Atif et al., 2021).

## 4 Empirical results

### 4.1 Descriptive statistics

Table 2 presents summary statistics for main variables before winsorizing. From *Raw Monetary* (the compensation per employee before the logarithmic transformation), the minimum value of the sample is ¥11,165.29 and the maximum is astonishing ¥9,933,697.88. In order to test whether this maximum is an outlier or a data error, we did a more in-depth statistical analysis of *Raw Monetary.* Specifically, among all firm-year observations, 2,904 observations are larger than the mean of *Raw Monetary* (151,940.96), and the mean of these observations is ¥272,006.4. In addition, there are 522 observations that are over ¥300,000, 232 that are over ¥400,000, and 113 that are over ¥500,000. This shows that the average salary of employees in listed firms in China is very impressive, and it varies a lot from firm to firm. Even after the logarithmic transformation (i.e., Monetary, one of the dependent variables of this study), the difference between its minimum (9.32) and maximum (16.11) is still very obvious. For another dependent variable, *NonMonetary*, some of the sample firms implemented none (Min = 0), while others implemented all eight of the non-monetary programs that were interested in this study (Max = 8). However, there is also a considerable disparity in the way that various listed firms conduct non-monetary activities (Mean = 4.72, Std. Dev. = 1.66). By looking at the independent variable *ShannonIndex*, it can be seen that its mean is 0.43, which indicates that the proportion of men on the boards of the sample firms is slightly higher than that of female directors on average but not completely dominant. But from its minimum value (0.11), it is clear that the phenomenon of male directors dominating the board exists strongly.

**Table 2:** Descriptive statistics of main variables.

VarName	Obs	Min	Mean	Median	P90	Max	Std. Dev.
Raw Monetary	8,973	11,165.29	151,940.96	119,472.52	252,636.63	9,933,697.88	242,510.66
Monetary	8,973	9.32	11.70	11.69	12.44	16.11	0.61
NonMonetary	8,973	0.00	4.72	5.00	7.00	8.00	1.66
ShannonIndex	8,973	0.11	0.43	0.43	0.62	0.69	0.14
Size	8,973	18.71	22.97	22.82	24.93	28.51	1.47
Leverage	8,973	0.01	0.48	0.49	0.73	1.70	0.20
ROA	8,973	−1.12	0.06	0.06	0.13	0.71	0.07
Property	8,973	0.00	0.57	1.00	1.00	1.00	0.50
SharesBalance	8,973	0.00	0.66	0.46	1.54	4.00	0.59
Duality	8,973	0.00	0.19	0.00	1.00	1.00	0.40
Independence	8,973	0.00	37.56	36.36	44.44	80.00	5.89

In terms of control variables, there is a significant difference between firm size (*Size*) and the number of independent directors (*Independence*) of the sample firms (Std. Dev. is equal to 1.47 and 5.89, respectively). Additionally, there is a substantial disparity between the profitability (*ROA* ranges from −1.12 to 0.71) and the overall performance is weak (mean of *ROA* is only 0.06). The sample firms also have a slightly higher proportion of state-owned than non-state-owned firms (the mean of *Property* is 0.57 and the median is 1.00). Moreover, the phenomenon of “the single-large shareholder” in the sample firms is more widespread and significant (the minimum of *SharesBalance* is 0 and the mean is 0.66). However, given its mean is 0.19 and median is 0, the CEO duality phenomena are not noteworthy in this study.

### 4.2 Correlation analysis and variance inflation factor test

Table 3 shows the Pearson correlations among the variables used in the baseline regression analysis. As expected, it can be seen that the independent variable (*ShannonIndex*) and the dependent variable (*Monetary* and *NonMonetary*) in this study are highly positively correlated (correlation coefficients are 0.053*** and 0.040***, respectively), which is in line with the theoretical analysis and research hypotheses 1a and 1b of this study. Table 4 displays the multicollinearity analysis of each major variable in this study. Multicollinearity among variables refers to the high correlation between the variables in the linear regression model, i.e., the dependent variable’s measure can be expressed by more than one independent variable, causing some overlap and crossover among the independent variables and inaccurate results from the linear regression model estimation (Skarmeas et al., 2014). We can see that multicollinearity does not exist among the main variables in this study from Table 4 because the maximum and mean VIF values of our variables are less than the usual threshold value of 10, i.e., the selection of each variable in this study satisfies the selection criteria.

**Table 3:** The Pearson correlation matrix.

	Monetary	NonMonetary	ShannonIndex	Size	Leverage	ROA	Property	SharesBalance	Duality	Independence
Monetary	1.000									
NonMonetary	0.196***	1.000								
ShannonIndex	0.053***	0.040***	1.000							
Size	0.377***	0.194***	−0.203***	1.000						
Leverage	0.099***	−0.003	−0.165***	0.517***	1.000					
ROA	−0.024**	0.040***	0.066***	−0.087***	−0.346***	1.000				
Property	0.145***	−0.014	−0.294***	0.307***	0.231***	−0.142***	1.000			
SharesBalance	−0.007	0.058***	0.095***	−0.090***	−0.104***	0.037***	−0.274***	1.000		
Duality	−0.023**	0.015	0.129***	−0.140***	−0.117***	0.074***	−0.313***	0.097***	1.000	
Independence	0.078***	−0.003	0.023**	0.102***	0.035***	−0.021**	−0.027**	−0.038***	0.083***	1.000

***, **, * denote statistical significance at the 1, 5, and 10% level, respectively.

**Table 4:** The variance inflation factor tests.

Variable	VIF	1/VIF	VIF	1/VIF
	Monetary		NonMonetary	
Leverage	1.57	0.636611	1.57	0.636611
Size	1.5	0.668426	1.5	0.668426
Property	1.36	0.734396	1.36	0.734396
ROA	1.16	0.859134	1.16	0.859134
Duality	1.12	0.891455	1.12	0.891455
ShannonIndex	1.12	0.895355	1.12	0.895355
SharesBalance	1.09	0.92015	1.09	0.92015
Independence	1.03	0.974701	1.03	0.974701
Mean VIF	1.24		1.24	

### 4.3 Baseline estimation

First, we examine the impact of board gender diversity on corporate employee benefits. Table 5 presents the results of the baseline regressions using OLS and Poisson models. All of the above specifications suggest that more gender-diverse boards have a significantly positive impact on the monetary benefits (*Monetary*) and non-monetary program implementation (*NonMonetary*) (at the 1 and 5% levels, respectively), indicating that the employee benefits in the Chinese A-listed firms were significantly improved under the higher board gender diversity, which was consistent with our hypotheses 1a and 1b. For instance, a one-percentage-point increase in the percentage of board gender diversity leads to an increase in monetary benefits of between 1.03 and 0.104 percent. Hence, the economic significance is also high. In addition to board gender diversity, firm size (*Size*) and ROA (*ROA*) also have a significantly positive relationship with monetary benefits and non-monetary program implementation. In contrast, leverage (*Leverage*) and shares balance (*SharesBalance*) have a significantly negative impact on monetary benefits, while board independence (*Independence*) significantly hinders non-monetary program implementation.

**Table 5:** Baseline estimation results: board gender diversity and employee benefits.

	(1)	(2)
	OLS	Poisson
	Monetary	NonMonetary
ShannonIndex	0.1040***	0.0586**
	(0.0350)	(0.0247)
Size	0.0856***	0.0405***
	(0.0047)	(0.0030)
Leverage	−0.1613***	−0.0353
	(0.0335)	(0.0232)
ROA	0.5609***	0.2815***
	(0.0947)	(0.0634)
Property	0.1441***	0.0062
	(0.0118)	(0.0080)
SharesBalance	−0.0172**	0.0099*
	(0.0083)	(0.0059)
Duality	0.0028	−0.0017
	(0.0121)	(0.0086)
Independence	0.0008	−0.0020***
	(0.0009)	(0.0006)
_cons	8.6589***	−0.3233**
	(0.1872)	(0.1306)
YearDum	Yes	Yes
IndustryDum	Yes	Yes
Observations	8,973	8,973
R-squared	0.438	/

(1) Robust standard errors in parentheses. (2) ***, **, * denote statistical significance at the 1, 5, and 10% level, respectively.

Following socialization theory, women have been conditioned to be more nurturing, compassionate, cooperative, and devoted to honing their interpersonal skills (Zelezny et al., 2000; Tunyi et al., 2023). They have also been socialized to have a high likelihood of participating in charitable activities and community service (Hillman et al., 2002). Therefore, female directors may help the board adopt a more democratic, participatory, and communal leadership style (Ben-Amar et al., 2017). Meanwhile, based on token theory (Kanter, 1977; Smith and Parrotta, 2018; Guldiken et al., 2019), the likelihood of appointing a second female to the board after the first one is much lower. More importantly, female directors are more likely to become a token and have inferior decision-making authority than male directors. Hence, female directors are more inclined to “please” staff by making immediate changes to policies (Kolpakov and Boyer, 2021), including compensation hikes, to strengthen their position on the boards since they must expend more effort on status-building than male directors (Bilimoria and Piderit, 1994; Smith and Parrotta, 2018). Meanwhile, according to Sittenthaler and Mohnen (2020), men’s performance is significantly higher in response to monetary incentives compared to non-monetary ones, and women’s performance is significantly higher in response to non-monetary incentives due to the feelings of appreciation and perceived performance pressure in a tournament setting. Therefore, except for the high-risk reforms in pay raising, the risk-averse character of female directors also makes them prone to implement some short-term, low-cost, and low-risk incentives like non-monetary rewards, which can also maintain the same level of job satisfaction.

### 4.4 Moderating effects of firm-level characteristics

Firm-level characteristics affect employee benefits. In this section, we investigate whether the relationship between board gender diversity and employee benefits would change under different firm-level characteristics: ownership structures, executive compensation, and institutional ownership.

#### 4.4.1 Moderating effects of ownership structure

Based on the prior literature, the ownership structure may affect firm employee benefits. To provide in-depth insights into these effects, our sample firms were classified as state-owned firms (SOEs) and non-state-owned firms (Non-SOEs) groups. Table 6 shows the regression results for the moderating effect of ownership structure, from which it can be seen that the coefficient for *ShannonIndex* was significantly positive at the 5% level in the SOE group, indicating that the higher board gender diversity of state-owned firms has a strong positive impact both on their monetary benefits and non-monetary program implementation. However, for the non-state-owned firms (Non-SOE group), the higher board gender diversity only shows a significant positive impact on their monetary benefits. This coincides with hypothesis 2 in the previous section.

**Table 6:** Moderating effect of ownership structure.

	(1)	(2)	(3)	(4)
	OLS	Poisson	OLS	Poisson
	SOEs group		Non-SOEs group	
	Monetary	NonMonetary	Monetary	NonMonetary
ShannonIndex	0.0984**	0.0864**	0.1462***	0.0135
	(0.0472)	(0.0340)	(0.0499)	(0.0358)
Size	0.0974***	0.0428***	0.0635***	0.0355***
	(0.0058)	(0.0038)	(0.0078)	(0.0047)
Leverage	−0.2353***	−0.0713**	−0.0179	0.0207
	(0.0441)	(0.0310)	(0.0518)	(0.0355)
ROA	0.7050***	0.2405**	0.5217***	0.2820***
	(0.1366)	(0.0962)	(0.1266)	(0.0830)
SharesBalance	−0.0870***	0.0040	0.0317***	0.0205***
	(0.0114)	(0.0091)	(0.0112)	(0.0079)
Duality	−0.0599***	−0.0022	0.0218	−0.0099
	(0.0211)	(0.0162)	(0.0143)	(0.0100)
Independence	−0.0005	−0.0014*	0.0027**	−0.0034***
	(0.0011)	(0.0008)	(0.0013)	(0.0009)
_cons	8.5255***	−0.2164	8.5839***	−0.3268*
	(0.2476)	(0.1792)	(0.1827)	(0.1911)
YearDum	Yes	Yes	Yes	Yes
IndustryDum	Yes	Yes	Yes	Yes
Observations	5,093	5,093	3,880	3,880
R-squared	0.428	/	0.482	/

(1) Robust standard errors in parentheses. (2) ***, **, *denote statistical significance at the 1, 5, and 10% level, respectively.

For state-owned firms, assuming corporate social responsibility is a higher priority than maximizing their economic interests. Therefore, compared with Non-SOEs, Chinese SOEs have been performing relatively better in terms of social responsibility. As employee benefit is a part of CSR, the commitment of SOEs to actively undertake and conscientiously fulfill is confirmed in the empirical results of this study. This finding was consistent with prior research (Li and Zhang, 2010). In addition, generally speaking, SOEs have a relatively larger firm size and a more comprehensive organizational system. Therefore, they are also comparatively better equipped to administer non-monetary program rewards than Non-SOEs. For non-state-owned firms, the maximization of shareholders’ profits and the sustainability of the firms are the ultimate goals of them (Li and Zhang, 2010). Compared to designing and implementing non-monetary programs through a large amount of human, material, and financial resources, the marginal benefits are much lower than direct pay rises. Additionally, Non-SOEs frequently lack the capability and ideal system needed to implement non-monetary projects in a systematic manner. Therefore, it is reasonable for female directors of Non-SOEs to choose the simpler monetary incentives as their employee-friendly policies.

#### 4.4.2 Moderating effects of executive compensation

To confirm the impact of the executive compensation differences on the employee benefits under board gender diversity, the sample firms were divided into higher and lower executive compensation firms according to the average value of the executive compensation grouped by industry. Table 7 shows the regression results of this moderating effect. We can see that the improvement impact on firm employee benefits due to board gender diversity was still significantly positive when the executive compensation was lower than the industry average in columns (3) and (4), but not in the higher executive compensation group (see columns (1) and (2)). This is in line with our hypothesis 2.

**Table 7:** Moderating effect of executive compensation.

	(1)	(2)	(3)	(4)
	OLS	Poisson	OLS	Poisson
	Higher Executive Compensation		Lower Executive Compensation	
	Monetary	NonMonetary	Monetary	NonMonetary
ShannonIndex	−0.0086	0.0379	0.2054***	0.0811**
	(0.0511)	(0.0334)	(0.0473)	(0.0362)
Size	0.0502***	0.0381***	0.1053***	0.0308***
	(0.0068)	(0.0041)	(0.0074)	(0.0051)
Leverage	−0.0738	0.0180	−0.2436***	−0.0611*
	(0.0544)	(0.0345)	(0.0432)	(0.0317)
ROA	0.3804**	0.2466***	0.4043***	0.2580***
	(0.1493)	(0.0898)	(0.1269)	(0.0950)
Property	0.1290***	0.0051	0.1788***	0.0159
	(0.0163)	(0.0105)	(0.0170)	(0.0124)
SharesBalance	−0.0149	0.0152**	−0.0194*	−0.0041
	(0.0120)	(0.0078)	(0.0115)	(0.0091)
Duality	−0.0279	0.0028	0.0452***	−0.0121
	(0.0179)	(0.0113)	(0.0162)	(0.0132)
Independence	−0.0005	−0.0026***	0.0023*	−0.0014
	(0.0012)	(0.0008)	(0.0013)	(0.0009)
_cons	8.9772***	−0.2210**	8.3100***	−0.3053*
	(0.1482)	(0.0920)	(0.2294)	(0.1642)
YearDum	Yes	Yes	Yes	Yes
IndustryDum	Yes	Yes	Yes	Yes
Observations	4,371	4,371	4,602	4,602
R-squared	0.382	/	0.434	/

(1) Robust standard errors in parentheses. (2) ***, **, * denote statistical significance at the 1, 5, and 10% level, respectively.

As mentioned earlier, when management has higher compensation, directors who are incentivized by compensation have lower conflicts and invisible agency costs with shareholders (Radu and Smaili, 2022), so they may prefer shareholder-friendly policies over more costly employee-friendly policies. In addition, similar to employees who are motivated to be more productive (Tunyi et al., 2023), the moral hazard of directors, including female directors, will be notably reduced after they are motivated by higher compensation, which makes them work much harder for shareholders to protect their interests than to motivate employees.

#### 4.4.3 Moderating effects of institutional ownership

Following the test approach for executive compensation moderating effects, we also divided the sample firms into higher and lower institutional ownership groups according to the average of the institutional ownership grouped by industry. Columns (1) and (2) in Table 8 show the regression results of the higher institutional ownership firms, and columns (3) and (4) indicate the results of the lower institutional ownership firms, which demonstrate a significant positive impact on both monetary and non-monetary benefits. This result confirms our hypothesis 2.

**Table 8:** Moderating effect of institutional ownership.

	(1)	(2)	(3)	(4)
	OLS	Poisson	OLS	Poisson
	Higher Institutional Ownership		Lower Institutional Ownership	
	Monetary	NonMonetary	Monetary	NonMonetary
ShannonIndex	0.0857*	0.0399	0.1577***	0.0837**
	(0.0479)	(0.0339)	(0.0515)	(0.0359)
Size	0.0717***	0.0391***	0.0884***	0.0373***
	(0.0061)	(0.0039)	(0.0084)	(0.0054)
Leverage	0.0434	−0.0414	−0.3228***	−0.0164
	(0.0458)	(0.0330)	(0.0496)	(0.0334)
ROA	0.6339***	0.2864***	0.4918***	0.3028***
	(0.1299)	(0.0909)	(0.1400)	(0.0900)
Property	0.1721***	0.0195*	0.1262***	−0.0074
	(0.0160)	(0.0111)	(0.0182)	(0.0124)
SharesBalance	−0.0654***	−0.0092	0.0086	0.0239***
	(0.0120)	(0.0087)	(0.0112)	(0.0083)
Duality	0.0074	0.0331***	0.0058	−0.0327***
	(0.0181)	(0.0123)	(0.0167)	(0.0118)
Independence	0.0006	−0.0021***	0.0014	−0.0022**
	(0.0011)	(0.0008)	(0.0014)	(0.0009)
_cons	8.9192***	−0.1899	8.4683***	−0.4949***
	(0.2480)	(0.1553)	(0.2126)	(0.1909)
YearDum	Yes	Yes	Yes	Yes
IndustryDum	Yes	Yes	Yes	Yes
Observations	4,871	4,871	4,102	4,102
R-squared	0.457	/	0.444	/

(1) Robust standard errors in parentheses. (2) ***, **, * denote statistical significance at the 1, 5, and 10% level, respectively.

This phenomenon may stem from the governance intervention and monitoring role of institutional investors. To extract their own private benefits, institutional investors may inhibit employee-friendly policies preferred by female directors and may also increase information asymmetry between firms and their non-investment stakeholders like employees by interfering with firms’ information disclosure (Buchanan et al., 2018), thus hindering firms’ engagement in social responsibility such as employee friendliness. Furthermore, the excessive intervention of institutional investors in corporate governance also lowers the incentive effect and integrity level of management and increases the moral hazard and adverse selection of them (Guiso et al., 2015), which makes management more inclined to achieve short-term profit objectives of firms rather than long-term talent pool building and corporate resilience shaping via employee-friendly policies.

### 4.5 The heterogeneous effect among the non-monetary benefits analysis

Based on our definition of *NonMonetary*, non-monetary benefits encompass eight different kinds of benefits programs, namely stock-ownership plans, retirement benefits, safety management systems, safety generation training, occupational safety certification, employee vocational training, employee communication channels, and other non-monetary programs except the above seven programs. To test whether there are differences between different non-monetary programs, this study constructs a binary variable about the implementation of each of the above-mentioned non-monetary programs. Each non-monetary program results in a logical regression when the binary variable associated with it is set to 1 when the non-monetary program is implemented or 0, respectively.

Table 9 shows the regression results for the eight non-monetary benefits mentioned above. It can be seen that board gender diversity has a significant positive impact at the 1% level for both the stock-ownership plans and occupational safety certification programs. In addition, board gender diversity likewise exhibits a significant boost at the 10% level for the safety generation training program.

**Table 9:** The heterogeneous effect among the non-monetary programs.

	(1)	(2)	(3)	(4)	(5)	(6)	(7)	(8)
	Logit							
	Stock-ownership Plans	Retirement Benefits	Safety Management Systems	Safety Generation Training	Occupational Safety Certification	Employee Vocational Training	Employee Communication Channels	Other programs
ShannonIndex	0.5579***	0.3317*	−0.0064	−0.3261*	0.6825***	−0.0145	0.0489	0.3379
	(0.2016)	(0.1871)	(0.1791)	(0.1754)	(0.2008)	(0.3111)	(0.1664)	(0.2176)
Size	0.0778***	0.1901***	0.1589***	0.1083***	0.1041***	0.2483***	0.1871***	0.0712***
	(0.0247)	(0.0235)	(0.0219)	(0.0219)	(0.0245)	(0.0384)	(0.0204)	(0.0270)
Leverage	0.3377*	−0.2302	−0.0423	−0.4224***	0.3880**	−0.1151	−0.5570***	−0.0525
	(0.1833)	(0.1697)	(0.1647)	(0.1637)	(0.1833)	(0.2844)	(0.1533)	(0.2011)
ROA	2.5148***	0.6814	0.7777*	−0.1765	1.3781***	1.0854	1.2535***	0.4823
	(0.4910)	(0.4660)	(0.4494)	(0.4420)	(0.4977)	(0.8262)	(0.4186)	(0.5574)
Property	−0.3200***	0.4301***	0.3309***	0.0386	−0.2315***	−0.3057***	0.1225**	−0.3055***
	(0.0635)	(0.0611)	(0.0571)	(0.0558)	(0.0622)	(0.1055)	(0.0529)	(0.0698)
SharesBalance	0.0343	−0.0110	0.0049	0.0094	−0.0219	0.4059***	0.1227***	−0.0479
	(0.0455)	(0.0446)	(0.0423)	(0.0408)	(0.0471)	(0.0856)	(0.0392)	(0.0509)
Duality	0.1747**	−0.0591	−0.1449**	−0.1331**	−0.0073	0.2487**	0.0786	−0.0438
	(0.0703)	(0.0649)	(0.0614)	(0.0608)	(0.0679)	(0.1224)	(0.0580)	(0.0779)
Independence	0.0018	−0.0012	−0.0005	−0.0126***	−0.0398***	0.0057	−0.0030	0.0006
	(0.0047)	(0.0045)	(0.0043)	(0.0041)	(0.0054)	(0.0075)	(0.0039)	(0.0054)
_cons	−1.5224**	−3.3114***	−5.7426***	−3.5969***	−2.6884***	−5.1607***	−6.7656***	−2.6536***
	(0.6052)	(0.5601)	(0.9085)	(0.9447)	(0.9148)	(1.0389)	(1.1480)	(0.8026)
YearDum	Yes	Yes	Yes	Yes	Yes	Yes	Yes	Yes
IndustryDum	Yes	Yes	Yes	Yes	Yes	Yes	Yes	Yes
Observations	8,954	8,958	8,973	8,953	8,966	8,801	8,970	8,953

(1) Robust standard errors in parentheses. (2) ***, **, * denote statistical significance at the 1, 5, and 10% level, respectively.

### 4.6 Robustness analysis

#### 4.6.1 Variable substitution

In Table 10, the female ratio of the board (*FemaleRatio*) was used as an alternative measure for board gender diversity to examine the positive relationship between gender diversity and employee benefits. The results were consistent with the main findings and supported hypotheses 1a and 1b.

**Table 10:** Robustness test 1: board gender diversity substitution.

	(1)	(2)
	OLS	Poisson
	Monetary	NonMonetary
FemaleRatio	0.2260***	0.0613*
	(0.0526)	(0.0359)
Size	0.0863***	0.0402***
	(0.0047)	(0.0030)
Leverage	−0.1582***	−0.0358
	(0.0334)	(0.0233)
ROA	0.5567***	0.2829***
	(0.0946)	(0.0634)
Property	0.1475***	0.0054
	(0.0118)	(0.0080)
SharesBalance	−0.0163**	0.0101*
	(0.0083)	(0.0059)
Duality	0.0024	−0.0015
	(0.0121)	(0.0086)
Independence	0.0007	−0.0020***
	(0.0009)	(0.0006)
_cons	8.6431***	−0.3032**
	(0.1864)	(0.1303)
YearDum	Yes	Yes
IndustryDum	Yes	Yes
Observations	8,973	8,973
R-squared	0.439	/

(1) Robust standard errors in parentheses. (2) ***, **, * denote statistical significance at the 1, 5, and 10% level, respectively.

#### 4.6.2 Time interval change

Despite the DEI (diversity, equality, and inclusion) campaigns’ present global popularity and the fact that more and more businesses are placing a greater emphasis on gender diversity on their boards, in terms of timing, a great deal of attention to gender diversity on the boards of modern firms in China began in 2012. In 2012, the Singapore Exchange (SGX) promoted having more female directors on boards. It established a secretariat for the Council for Board Diversity and its predecessor, the Diversity Action Committee. Subsequently, this reform movement spread to China. Chinese listed firms began to actively promote gender diversity on their boards. Therefore, in the second robustness test of this study, we change the time window of our sample from 2012 to 2021. By selecting more recent sample data, we may add more thorough information regarding gender diversity and bring our results up to date with the market as it stands. As can be seen from the results in Table 11, the positive impact of board gender diversity on employee benefits remained the same when the time window changed to 2012–2021. Hypotheses 1a and 1b of this study are further robustly tested.

**Table 11:** Robustness test 2: time interval change.

	(1)	(2)
	OLS	Poisson
	Monetary	NonMonetary
ShannonIndex	0.0689*	0.0533**
	(0.0358)	(0.0263)
Size	0.0789***	0.0399***
	(0.0048)	(0.0032)
Leverage	−0.1443***	−0.0150
	(0.0348)	(0.0252)
ROA	0.5974***	0.3486***
	(0.0998)	(0.0673)
Property	0.1311***	−0.0006
	(0.0124)	(0.0085)
SharesBalance	−0.0063	0.0087
	(0.0088)	(0.0065)
Duality	0.0146	−0.0089
	(0.0129)	(0.0090)
Independence	0.0015	−0.0019***
	(0.0009)	(0.0006)
_cons	9.0736***	0.2917***
	(0.1133)	(0.0799)
YearDum	Yes	Yes
IndustryDum	Yes	Yes
Observations	7,371	7,371
R-squared	0.384	/

(1) Robust standard errors in parentheses. (2) ***, **, * denote statistical significance at the 1, 5, and 10% level, respectively.

#### 4.6.3 Lagged independent variable and model change

Female directors and board characteristics require some time to influence firm policies (Atif et al., 2021), including changes in employee monetary benefits. However, in terms of the nature of monetary and non-monetary benefits, as well as the overall strategic layout of the firms, changes to monetary incentives affect firms more profoundly and broadly than changes to non-monetary benefits. This is because altering the firm’s total compensation design, which involves almost all employees has a higher and long-term impact on operating costs and final earnings than implementing non-monetary programs. As a result, firms are frequently more cautious when reforming the compensation structure, as well as taking a long time to decide and evaluate the pay raise. Therefore, we use one-year lagged independent variables by replacing the contemporaneous variables to mitigate the endogeneity concerns in the relations between gender diversity and monetary benefits. As can be seen from the result in column (1) of Table 12, board gender diversity with a one-year lag still has a significant positive contribution to firms’ monetary benefits at the 1% level.

**Table 12:** Robustness test 3: lagged independent variable and model change.

	(1)	(2)
	One-year Lagged OLS	Tobit
	Monetary	NonMonetary
Lag.ShannonIndex	0.1032***	
	(0.0381)	
ShannonIndex		0.2632**
		(0.1165)
Size	0.0863***	0.1915***
	(0.0051)	(0.0144)
Leverage	−0.1480***	−0.1579
	(0.0368)	(0.1082)
ROA	0.5405***	1.4193***
	(0.1023)	(0.3009)
Property	0.1429***	0.0292
	(0.0128)	(0.0378)
SharesBalance	−0.0225**	0.0509*
	(0.0090)	(0.0281)
Duality	−0.0014	−0.0112
	(0.0135)	(0.0408)
Independence	0.0015	−0.0090***
	(0.0009)	(0.0028)
_cons	8.2048***	−2.6449***
	(0.1469)	(0.4318)
var(e.NonMonetary)	/	2.0569***
	/	(0.0307)
YearDum	Yes	Yes
IndustryDum	Yes	Yes
Observations	7,377	8,973
R-squared	0.425	/

(1) Robust standard errors in parentheses. (2) ***, **, * denote statistical significance at the 1, 5, and 10% level, respectively.

For non-monetary benefits, we did not utilize the same one-year lag of the independent variables to perform the robust test because, except for stock-ownership plans and retirement benefits, the remaining non-monetary programs are generally short-term reforms with a relatively small scope of influence. Since *NonMonetary* is an integer value taken from [0, 8], i.e., *NonMonetary* is a trailing variable with 0 as the lower bound. Therefore, we use the Tobit model to conduct a regression to assess the effect of gender diversity on non-financial benefits in light of this data distribution. The result in column (2) of Table 12 shows that the conclusion of the significantly positive promotion of gender diversity for non-monetary benefits remains valid.

#### 4.6.4 Instrument variable analysis

Concerns about the potential endogeneity of female board presence are frequently raised in the gender diversity research (Khatib et al., 2021). For instance, firms with better employee benefits are more likely to have a greater diversity level on the boards, and even the nomination and appointment of female directors can be a reflection of the company’s (female) employee benefits. Hence, our main findings might therefore be the product of correlation rather than causality. Therefore, we address this endogeneity concerns by adopting an instrumental variable (IV) approach and estimating the regressions via Two-stage Least Squares (2SLS) to extract the exogenous component from board gender diversity. We then use the latter to explain employee benefits. The challenge in using 2SLS is the identification of exogenous IVs that do not have a direct relationship with the dependent variable (Atif et al., 2021).

We use the female-to-male workforce participation ratio (*Female–Male-Ratio*) as an IV for board gender diversity. The labor force here is defined as the economically active population aged 15 years and over. The IV is computed as the female participation ratio divided by the male participation ratio at the national level. This data was collected from the World Bank. Similar to Chen et al. (2019) and Atif et al. (2021), We chose this IV because years with a greater female-to-male participation ratio are more likely to provide excellent female directors because there is a wider pool of applicants, and should consequently have a higher percentage of female directors. In contrast, there is little evidence, if any, that suggests that the female-to-male participation ratio of the nation affects the firm’s employee benefits. Hence, we expect our IV (*Female–Male-Ratio*) to be positively correlated with board gender diversity (*ShannonIndex*).

For the validity test of this IV approach, the F statistical value in the Cragg-Donald weak identification test is very high (31.070 and 1563.254 for *Monetary* and *NonMonetary* respectively), and its *p*-value is 0.000, which rejects the null hypothesis of the weak tool (Cragg and Donald, 1993; Stock and Yogo, 2005). Table 13 shows the results of monetary benefits and non-monetary program building from the 2SLS estimation, which, after controlling for possible gender diversity reserve endogeneity, remained similar to those from our main regression analysis that suggests a significant positive relationship between the gender diversity of the board and employee benefits.

**Table 13:** Robustness test 4: instrumental variable analysis.

	(1)	(2)
	2SLS	2SLS
	Monetary	NonMonetary
Female–Male-Ratio	4.8451***	4.6134***
	(0.1916)	(0.4143)
Size	0.2229***	0.3782***
	(0.0073)	(0.0157)
Leverage	−0.2326***	−0.9013***
	(0.0534)	(0.1154)
ROA	−0.3409**	0.2408
	(0.1550)	(0.3352)
Property	0.4064***	0.1457***
	(0.0232)	(0.0501)
SharesBalance	0.0158	0.1349***
	(0.0149)	(0.0322)
Duality	−0.0014	0.0362
	(0.0223)	(0.0481)
Independence	0.0005	−0.0120***
	(0.0015)	(0.0033)
_cons	4.3822***	−5.2452***
	(0.2009)	(0.4344)
YearDum	Yes	Yes
IndustryDum	Yes	Yes
Observations	8,973	8,973

(1) Robust standard errors in parentheses. (2) ***, **, * denote statistical significance at the 1, 5, and 10% level, respectively.

### 4.7 Summary of empirical results

**Figure 2:**
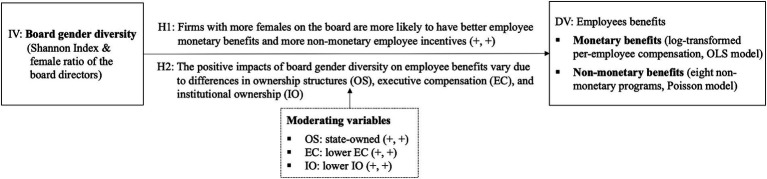
Summary of hypotheses, measures, and results.

Figure 2 provides a succinct overview of our empirical setting and results, summarizing key findings for each hypothesis. Specifically, based on the OLS model and Poisson model, H1a and H1b proposed in this study are fully supported. We also use a measure substitution of board gender diversity, sample time interval change, lagged independent variable, model change, and instrumental variable to further confirm the robustness of the main regression results. Furthermore, in exploring the impact of firm-level characteristics on the above relations, this study finds that the board mentioned above gender diversity contributes more significantly to employee benefits when the firm is a state-owned firm, or when the total compensation of management is lower, or when it has fewer institutional ownership.

## 5 Conclusion

This study examined the impact of board gender diversity on employee benefits such as employee compensation incentives and non-monetary benefit programs. The empirical results based on the 2006–2021 A-share listed firms in China show that the more diverse gender construct increased the employee average salary and the likelihood of the implementation of non-monetary programs, such as stock-ownership plans, retirement benefits, and occupational safety certification. Additionally, we discover that this beneficial association is more prominent in state-owned firms, as well as in cases with lower executive compensation and less institutional ownership. We also use a measure substitution of board gender diversity, sample time interval change, lagged independent variable, and model change to further confirm the robustness of the main regression results. In addition, to account for possible endogeneity concerns, the two-stage least squares model was used on our instrumental variable, the female-to-male workforce participation ratio, from which the robustness of the results was proven. Our findings align with the research of Sittenthaler and Mohnen (2020), which investigated the distinct predispositions exhibited by individuals of different genders concerning both monetary and non-monetary benefits. However, our emphasis lies in examining the viewpoint of benefit policymakers, thereby providing a more nuanced understanding of how to foster policymaking that is oriented toward promoting employee well-being and prioritizing a people-centered approach at its origin. This result may stem from the more sensitive, supportive, empathetic, inclusive, and ethical leadership style of female directors relative to their male counterparts, or it may be since female directors face more barriers and bottlenecks in advancement, which motivates them to adopt more employee-friendly policies like pay incentives and training opportunities for other potential employees. They may also work harder to uphold their position on the board through these employee-friendly policies.

The global focus on Diversity, Equity, and Inclusion (DEI) programs and the heightened attention toward female directors have spurred extensive research on the economic implications of gender diversity in corporate governance. Despite this, female representation on boards remains notably insufficient. Our study contributes to the literature on board gender diversity and employee relationship management by revealing that female directors exhibit a higher likelihood of championing both monetary and non-monetary employee-friendly benefits. This dual enrichment of literature not only highlights the positive impact of gender diversity on employee welfare but also provides nuanced insights and implications for corporate management and business practices. The observed positive associations, especially in state-owned firms, and the firms with lower executive compensation or less institutional ownership, accentuate the need for tailored diversity initiatives in specific organizational contexts. Recognizing gender diversity as a strategic imperative for sustainable growth, business leaders and boards should appreciate its potential to foster inclusive corporate cultures. As the DEI movement gains momentum globally, businesses are encouraged to integrate gender diversity into their strategic agendas, acknowledging its capacity to cultivate a more inclusive and employee-centric corporate ethos.

However, there are also three potential limitations of this study. First, although this study develops the mechanism for board diversity boosting employee remuneration incentives and non-monetary advantages at the theoretical level, it does not utilize an empirical model to test this underlying relationship, which is mainly limited by the data collection. For example, according to Sittenthaler and Mohnen (2020), the feelings of appreciation and perceived performance pressure in a tournament setting may explain why men and women respond differently to various kinds of benefits. However, collecting data on ‘feelings’ is primarily conducted through survey questionnaires and structured interviews which presents considerable challenges if conducting these surveys across a large number of listed companies in China. Meanwhile, there is a lack of a standardized or well-established questionnaire specifically designed to measure the ‘feelings’ indicator, adding complexity to the data collection process. Second, even the Shannon index and the proportion of female directors are both used in this study to measure board gender diversity, however previous research on board gender diversity indicates that the number of female directors also matters (Guldiken et al., 2019). Some literature contends that when there are only one or two women on a board, those women serve more as symbolic figures than real decision-makers. Female directors perform their duties as directors when there are three or more of them on the board of directors, but the heterogeneity in the number of female directors has not yet been examined in this study. Last, we leverage the compensation payable to employees from the balance sheet to calculate the monetary benefits which may result in double counting between monetary and non-monetary benefits. According to “Chinese Accounting Standards No.9-Employee Compensation” issued by the Ministry of Finance of China, the non-monetary benefits that are part of employee compensation should be recognized as employee compensation at fair value. Therefore, retirement benefits, etc. presented in this study may be double-counted as monetary benefits. However, in terms of data availability, the current public data of listed companies also does not break down the benefits in the compensation payable to employees, so this becomes a possible limitation of this paper. But from the perspective of the non-monetary benefits, except for stock-ownership plans, retirement benefits, and other non-monetary programs, the other rest five non-monetary programs are unlikely to be double-accounted for in monetary benefits. At the same time, from the methodology of this study, non-monetary benefits are measured by integer counts and regressed using a Poisson model in which the coefficients of independent variables express the multiplicative effect of changes in gender diversity on the incidence of non-monetary events, while the coefficients in OLS regression model for monetary benefits indicate the amount of change in monetary remuneration when the independent variable gender diversity changes by one unit. Thus, the inconsistent focus of the two different models relatively alleviates the limitation of possible double-measurement. Therefore, future studies can begin by looking at the potential mechanisms underlying female directors’ support for employee-friendly policies and confirming, through the collecting of more precise data, if the specific number of female directors does play a significant and symbolic role in employee-friendly policies. Also, if possible, the future study could have a data breakdown to differentiate what part of the monetary remuneration is for the monetary salary package and what part is for non-monetary benefits.

## Data availability statement

The original contributions presented in the study are included in the article/Supplementary material, further inquiries can be directed to the corresponding author.

## Author contributions

YL: Data curation, Formal analysis, Methodology, Software, Writing – original draft, Writing – review & editing. YT: Conceptualization, Investigation, Supervision, Writing – original draft, Writing – review & editing. YY: Conceptualization, Funding acquisition, Investigation, Supervision, Writing – original draft, Writing – review & editing.
